# Integrating QTL mapping and GWAS to decipher the genetic mechanisms behind the calcium contents of *Brassica napus* shoots

**DOI:** 10.3389/fpls.2025.1565329

**Published:** 2025-04-10

**Authors:** Yanan Xiang, Feng Chen, Rui Shi, Tinghai Yang, Wei Zhang, Xiaoying Zhou, Chunyun Wang, Chengming Sun, Sanxiong Fu, Xiaodong Wang, Jiefu Zhang, Yue Shen

**Affiliations:** ^1^ School of Food and Biological Engineering, Jiangsu University, Zhenjiang, China; ^2^ Institute of Industrial Crops, Jiangsu Academy of Agricultural Sciences, Key Laboratory of Cotton and Rapeseed, Ministry of Agriculture and Rural Affairs, Nanjing, China

**Keywords:** *Brassica napus* shoots, calcium concentration, QTL mapping, GWAS, candidate genes

## Abstract

*Brassica napus* is an important oil crop worldwide, and its shoots are rich in vitamin C, calcium, and selenium. Functional oilseed-vegetable-dual-purpose varieties can increase the subsidiary value of *B. napus*. Consumption of high-calcium *B. napus* shoots can effectively help provide essential elements to the human body. To investigate the genetic mechanisms underlying the calcium concentrations of *B. napus* shoots, quantitative trait loci (QTL) mapping, using a population of 189 recombinant inbred lines, and a genome-wide association study, using an association panel of 202 diverse accessions, were performed. A total of 12 QTLs controlling calcium content were identified using the recombinant inbred line population in five environments. Among them, *qCaC.22GY-A05-1* was considered the major QTL, with a phenotypic variation of 10.10%. In addition, 228 single nucleotide polymorphisms significantly related to calcium content were identified using the genome-wide association study in six environments, and they were distributed on all of the chromosomes, except A10. Finally, 10 candidate genes involved in regulating calcium absorption and transport in *B. napus* shoots were identified. However, no overlapping intervals were found through a comprehensive analysis of the two datasets. These results provide valuable information for understanding the genetic control of calcium concentration in *B. napus* shoots.

## Introduction

Rapeseed (*Brassica napus*, AACC, 2n = 38) is the third-largest oil crop in the world (Hu et al., 2021). In recent years, to increase its economic value rapeseed cultivars have been developed as sources of flowers, feed, and vegetables (Shi et al., 2023). The production of oilseed-vegetable-dual-purpose (OVDP) varieties can meet the double demand of the market for edible oil and vegetables (Wu et al., 2021). Picking *B. napus* shoots can promote secondary branching and increase the total silique number (Huang et al., 2014). This does not result in a significant decrease in seed yield, thereby increasing the economic value (Zhu et al., 2020). *B. napus* shoots have a high edible value and are rich in nutrients such as vitamin C, calcium (Ca), selenium (Se), and zinc (Zn) (Fu et al., 2022). *Brassica* vegetables, such as kale and broccoli, contain high levels of vitamins, minerals, and other bioactive substances that have antibacterial, anti-inflammatory, and anti-cancer effects (Podsędek, 2007; Mandrich and Caputo, 2020; Cicio et al., 2023; Garcia-Ibañez et al., 2024). *B. napus* belongs to the *Brassicaceae* family, consequently, its shoots have the potential to develop into functional vegetables. The yields and income changes resulting from growing OVDP varieties have been studied, but less attention has been paid to their nutritional qualities. At present, Se-rich *B. napus* varieties have been cultivated to enhance immunity (Huang et al., 2022), but no high-Ca *B. napus* OVDP varieties have been cultivated.

As the most abundant mineral element in the human body, Ca participates in various metabolic activities and is essential for maintaining health (Enders et al., 2020). Deficiencies in Ca can cause disease, and humans mainly supplement daily Ca intake by consuming plant-based foods, which may not meet nutritional requirements. The development of high-Ca vegetables will help meet consumer’s daily Ca needs, resulting in a population with a more balanced nutrition (Ma et al., 2023). Additionally, Ca is an essential plant nutrient element that plays a central role in growth, development, and responses to environmental stress. It is a structural component of cell walls and membranes, and a second messenger in cells (Thor, 2019). Calcium in the soil is passively and actively absorbed by plant roots in the form of ions, which are transported upward to stems and leaves under the action of transpiration or Ca-ATPases, and the intracellular redistribution is completed by channel and transporter proteins (White et al., 2002; DalCorso et al., 2014; Torres et al., 2024). Thus, there are many factors affecting the accumulation of Ca in *B. napus* during its sprout stage, and studies have mainly focused on increasing Ca concentration through different external conditions. However, a single external factor has limited influence on Ca concentration (CaC) (Yuan et al., 2018).

The CaC of a plant is a complex quantitative trait controlled by multiple genes and easily affected by environmental conditions (Jaiswal et al., 2019). Quantitative trait locus (QTL) mapping has been widely applied to dissect the genetic bases of mineral elements in crops (Ma et al., 2017; Wang et al., 2017). Genome-wide association studies (GWASs) are another useful method to study complex quantitative traits, and have the advantage of detecting the genetic structure of traits without the need for population construction (Saini et al., 2021). These studies have been widely used to identify candidate genes for quantitative vegetable traits, such as in soybean and pepper (Haile et al., 2023; Jo et al., 2023). In *B. napus*, GWASs have been used to investigate seed yield (Liu et al., 2022), seed oil content (Zhao et al., 2022), silique length (Wang et al., 2021), and seed fatty acids (Gazave et al., 2020). Additionally, GWAS have been used to dissect the accumulation of minerals in other crops, such as pearl millet (Singh et al., 2024). Integrating GWASs and QTL mapping provides an effective method to understand the genetic structures of quantitative traits. The combination has been widely applied to genetic analyses of maize yield (Wang et al., 2023), soluble solid content in melon (Yan et al., 2023), and cadmium accumulation in maize leaves (Zhao et al., 2018). In *B. napus*, two traits, soluble solid content (Wu et al., 2021) and crude fiber, have been studied using QTL mapping and GWAS combined (Shi et al., 2023, Shi et al., 2024).

In this study, QTL mapping and GWAS were performed to obtain loci controlling CaC in *B. napus* shoots using phenotypic data in multiple environments. This study aimed to increase the understanding of genetic mechanisms related to CaC in *B. napus* shoots at the stem elongation stage and to provide excellent germplasm resources for breeding high-Ca OVDP varieties.

## Materials and methods

### Experimental materials

In the present study, a recombinant inbred line (RIL) population, containing 189 lines and named the AH population (Wang et al., 2015), and an association panel consisted of 202 diverse accessions, named the CF population (Shi et al., 2024), were used for QTL mapping and GWAS of the loci associated with CaC. All the materials were planted in Nanjing City, Jiangsu Province, and Guiyang City, Guizhou Province, China. The AH population was planted in Guiyang for two consecutive years (2020–2021) and Nanjing for three straight years (2020–2022), and the CF population was planted in Nanjing and Guiyang for three straight years (2020–2022). The two populations were planted in October, and the shoots were harvested in the following March. According to planting location and harvesting time, experimental materials are denoted as 21NJ, 22NJ, 23NJ, 21GY, 22GY, and 23GY. All of the experimental materials were used in a completely randomized block design. The single experimental plot consisted of two rows, with 20 plants per row and 40 cm between the rows. Field management practices adhered to local agricultural production standards (Sun et al., 2018). According to the national agricultural industry standard ‘Grades and specification of flowering Chinese cabbage’, three plants of nonflowering and uniform growth from *B. napus* shoots were harvested at the stem elongation stage. They were approximately 20 cm in length and of uniform thickness (Shi et al., 2024).

### Calcium element detection

The *B. napus* shoots were treated in an electric thermostatic air-drying oven (DGG-9240A, Senxin, Shanghai, China), dried at 105°C for 15 min, followed by drying at 80°C to constant weights. The dried samples were crushed in a grinder, passed through a 60-mesh screen, and maintained at low temperatures (Nerdy, 2018).

In accordance with the method of Saleem et al (Saleem et al., 2021), the determination of mineral elements were as follows: 0.25 g of sample was accurately weighed and placed into a digestion tube. Then, 8 mL of concentrated nitric acid and 2 mL perchloric acid were added, the tubes were sealed, and the samples were mixed. They were left standing for 24 h. A variable temperature digestion process of 100 °C for 1 h, 130 °C for 3 h, and finally 180 °C for 2.5 h was carried out, and samples were then allowed to naturally cool to room temperature. Each reaction sample solution was poured into a 25 mL test tube, washed with a small constant volume of nitric acid solution (1%) four times, then constant volume to 25ml. Finally, the solution to be tested was obtained by filtration.

Atomic absorption spectroscopy (AA-7000, Shimadzu Corporation, Kyoto, Japan) was used to measure the total CaC in samples. A standard curve was constructed using a mixed standard product. After diluting the solution to be measured by 20 times, the calcium concentration in the sample solution was detected. The calculation formula was as follows:


$$\text{CaC}\;(\text{mg}/\text{g})=[(\text{C}\times 20\times \text{V})/\text{m}]\times 10^{-6}$$

where m represents the weighed sample, V represents the constant volume, and C represents the Ca concentration of the sample.

### QTL mapping

A high-density linkage map was constructed for the AH population, and it contained 11,458 single nucleotide polymorphisms (SNPs) and 57 simple sequence repeat markers, covering a genome length of 2,027.53 cM, with an average distance of 0.72 cM between markers (Wang et al., 2015). The QTL analysis of CaC in *B. napus* shoots was performed using the composite interval mapping method of Windows QTL Cartographer 2.5 software (Wang et al., 2015). The LOD threshold was determined by 1,000 permutation tests (*P* ≤ 0.05) for each trait, and QTLs with LOD values greater than the threshold were considered as putative QTLs. The QTLs were named following the description of Wang et al. (Wang et al., 2015). The name begins with the letter ‘*q*’, followed by the character, environment, and chromosomal number. Serial numbers were added to distinguish whether multiple significant QTLs were detected on the same chromosome in the same environment, such as *qCaC.21NJ.A1-1*.

### GWAS analysis

A DNA library of the CF population was constructed and sequenced, with a sequencing depth of ~20×. A total of 6,093,649 SNPs and 996,252 InDels were detected in the whole genome of the GWAS population, with 2,719,348 and 3,352,074 SNPs in the A and C subgenomes, respectively (Shi et al., 2024). The mixed linear model in the TASSEL5.1 software (trait analysis by association, evolution and linkage) was used for the GWAS analysis (Bradbury et al., 2007), and two correction modes, kinship and population stratification, were added. The significance threshold was determined using the Genetic Type I error calculator, and 1,454,769 highly-effective sites were obtained (Yang et al., 2011; Li et al., 2012; Zhou and Stephens, 2012). The average linkage disequilibrium decay distance for the whole genome was 90.3 kb when *r* ^2^ = 0.2; when *r* ^2^ was decreased to half of the maximum value (*r* ^2^ = 0.345), the decay distance was 4 kb (Shi et al., 2024). The Manhattan and QQ plots were constructed using the qqman package in R software (Turner, 2018; Wu et al., 2021).

### Candidate gene prediction

In AH population, based on the resequencing results, all the variation sites between parents were identified (Yu et al., 2021). Then, using the SNP probe sequence on the genetic map, the markers on both sides of the QTL interval were mapped to the ‘Darmor-bzh’ reference genome using Blast (E-value ≤ 1e-10) software (Chalhoub et al., 2014). Then, SNPs and InDels within the QTL interval, and SNPs causing non-synonymous mutations and InDels causing frameshift mutations, were identified. Finally, for SNPs with non-synonymous mutations and InDels with frameshift mutations, candidate genes related to the accumulation of calcium were screened based on *Arabidopsis* gene function annotations.

In addition, in CF population, by analyzing the gene and protein sequences of the ‘ZS11’ reference genome within the linkage disequilibrium (90.3 kb) range, the gene coding sequences of significant sites were extracted. Blast2GO (v3.3) was used for the enrichment analysis of all the genes, and the whole genome sequence was used as a reference. Then, functional annotation information on candidate genes was obtained, and candidate genes related to CaC were screened based on the functional gene annotations (Yang et al., 2024).

## Results

### Statistical analysis of phenotypic data

**Figure 1:**
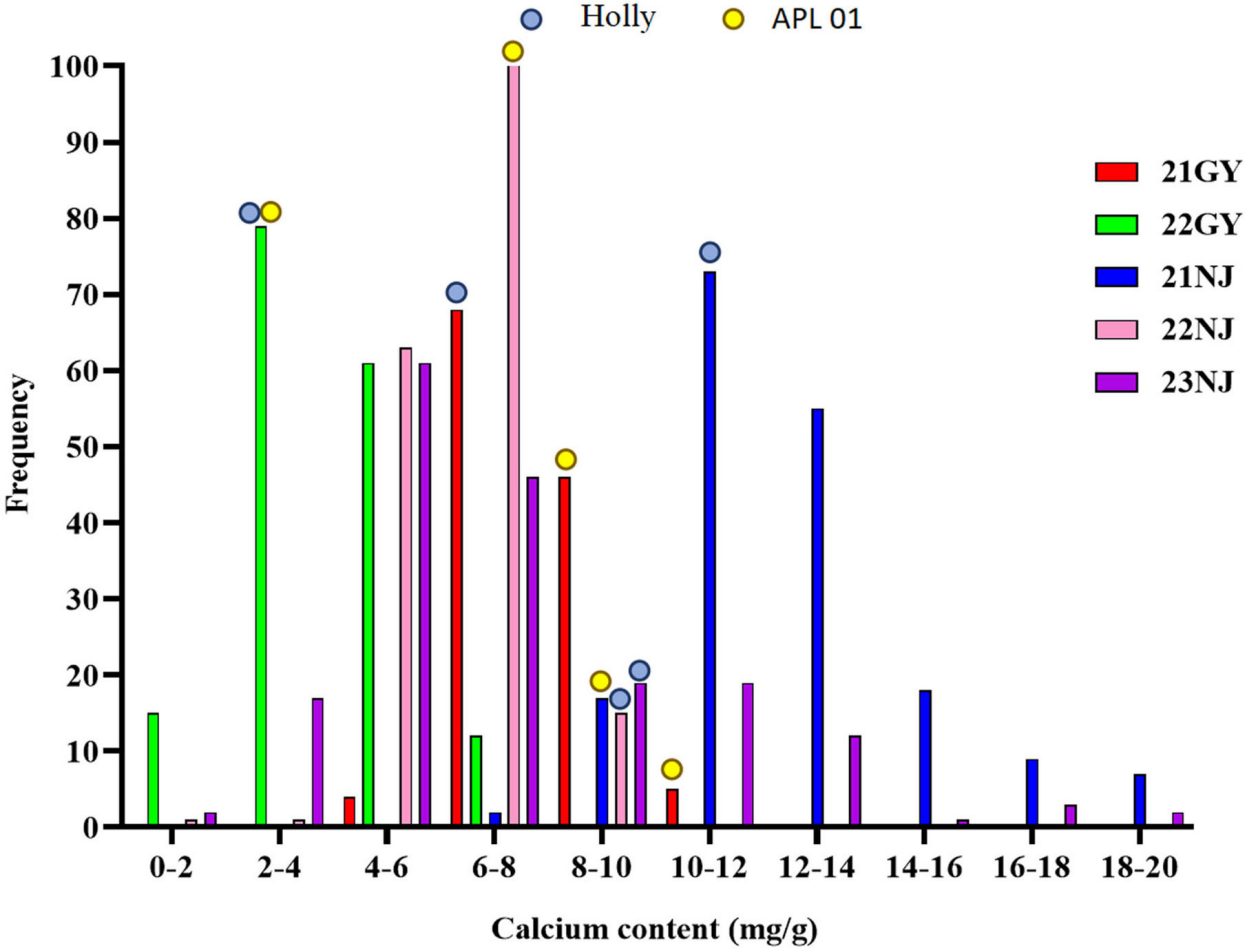
Frequency distributions of five environmental phenotypes in the AH recombinant inbred lines (RIL) population. Blue dots represent the calcium concentration of the male parent ‘Holly’ in five environments; Yellow dots represent the calcium concentration of the female parent ‘APL01’ in five environments.

**Figure 2:**
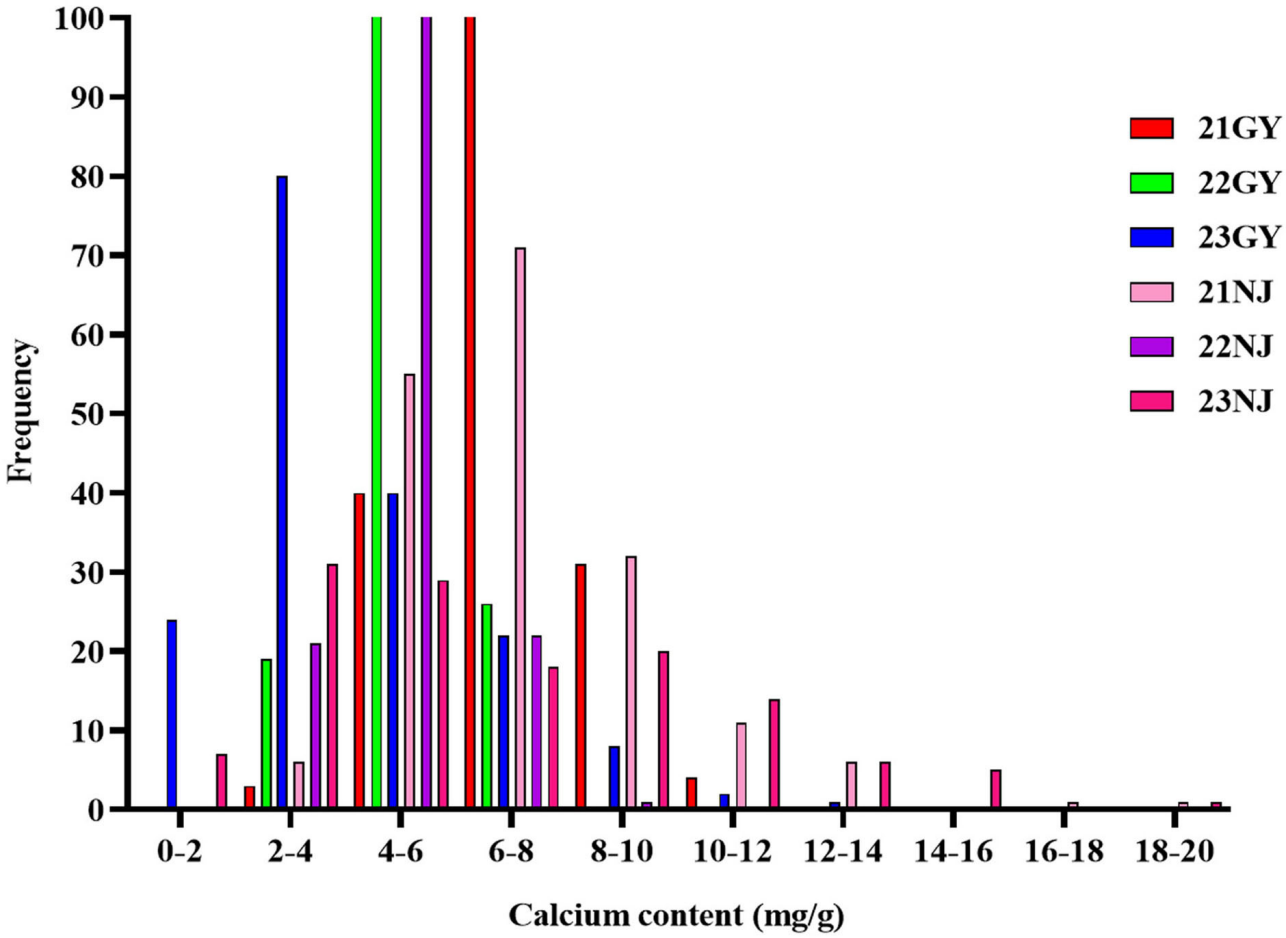
Frequency distributions of six environmental phenotypes in the CF population.

In this study, the CaC of the parents and the AH population lines in five environments (**Table 1**; **Figure 1**), and the CF population lines in six environments (**Table 2**; **Figure 2**), were analyzed. The CaC in the AH and CF population lines were normally distributed, indicating that CaC was a quantitative trait controlled by multiple genes.

**Table 1:** Analysis of Ca concentration in the AH population.

Environment	Parents		RIL Lines				
	Holly	APL01	Min	Max	Mean	SD	CV (%)
21GY	7.94	10.04	4.90	11.02	7.80	1.13	14.50
22GY	3.82	2.55	1.38	5.18	2.92	0.71	24.20
21NJ	10.30	9.04	7.52	37.31	12.63	3.46	27.40
22NJ	9.29	7.85	0.05	9.89	6.47	1.17	18.20
23NJ	9.04	7.85	1.54	20.67	7.24	3.37	46.50

Min, minimum value; Max, maximum value; SD, standard deviation; CV, coefficient of variation.

This table presents the ranges, mean values, SD, and CV of Ca concentrations in the AH population across different environments (year, planting area). The mean values (*n* = 3) for the parental lines (‘Holly’ and ‘APL01’) are also provided.

**Table 2:** Analysis of Ca concentration in the CF population.

Environment	Min	Max	Mean	SD	CV (%)
21GY	5.12	13.52	8.92	1.42	15.93
22GY	3.31	8.09	5.06	0.95	18.73
23GY	0.95	12.17	4.06	2.13	52.39
21NJ	3.14	19.71	7.23	2.35	32.56
22NJ	3.09	8.59	5.03	0.84	26.74
23NJ	0.67	18.15	6.69	3.81	57

Min, minimum value; Max, maximum value; SD, standard deviation; CV, coefficient of variation.

This table presents the ranges, mean values, SD, and CV of Ca concentrations in the CF populations across different environments (year, planting area).

In the AH population, CaC values of the male parent ‘Holly’ and female parent ‘APL01’ exhibited significant differences across individual environments, indicating that synergistic alleles were distributed in both parents (**Table 1**). There were significant differences in CaC among different lines. In the 21NJ environment, the minimum CaC value in the AH population was 7.52 mg/g, and the maximum CaC value was 37.31 mg/g (**Table 1**). In the 23NJ environment, the CaC in CF population ranged from 0.67 to 18.15 mg/g (**Table 2**).

The coefficients of variation among AH lines ranged from 14.5% to 46.5%, with an average of 26.16%, whereas the coefficients of variation among CF population lines ranged from 15.93% to 57%, with an average of 33.89%. This suggested that the phenotypic traits of the two populations changed significantly in different years and areas.

### QTL mapping of the AH population

The QTL mapping of CaC in the AH population was performed using the phenotypic data from five different environments. A total of 12 QTLs were detected, which were located on A1, A3, A5, A7, A9, and C7 (**Table 3**). Among the 12 QTLs, the phenotypic variance (PV) values explained by a single QTL ranged from 1.67% to 10.10%, and the additive effect ranged from −1.12 to 0.80. Four QTLs related to CaC were identified in the 21GY environment, of which three QTLs were located on chromosome A1 and the other QTL on chromosome C7. In the 22NJ environment, only one QTL was identified on chromosome A9. QTL *qCaC/22GY-A5-1* located on chromosome A5, with a PV value of 10.10%, was considered the major QTL conducive to improving CaC traits of *B. napus* shoots. However, no QTL was repeatedly detected in multiple environments, indicating that the environment significantly affected the CaC.

**Table 3:** The single QTL of Ca concentration in the AH population was detected in five environments.

Chr	QTL	Peak	Marker	CL (cM)	LOD	ADD	R^2^ (%)	Environment
A1	*qCaC.21GY.A1-1*	95.71	Bn-A01-p5787591-Bn-A01-p6199422	94.8-98.3	3.04	-0.34	8.36	21GY
A1	*qCaC.21GY.A1-2*	102.31	Bn-A01-p3789302-Bn-A01-p5787591	101.9-102.6	2.72	-0.33	7.86	21GY
A1	*qCaC.21GY.A1-3*	105.11	Bn-A01-p3487320-Bn-A01-p5787591	105-106.4	0.50	-0.32	1.67	21GY
A3	*qCaC.23NJ.A3-1*	47.31	Bn-A03-p16275650-Bn-scaff_17298_1-p705887	47-47.9	4.29	-1.04	9.08	23NJ
A3	*qCaC.23NJ.A3-2*	90.51	Bn-A03-p7887578-Bn-A03-p9497821	90.3-91	2.56	+0.80	5.35	23NJ
A5	*qCaC.22GY.A5-1*	25.31	Bn-A05-p21029850-Bn-A05-p21163226	24.9-25.5	4.62	-0.26	10.10	22GY
A5	*qCaC.22GY.A5-2*	42.61	Bn-A05-p19001293-Bn-A05-p19224833	41.6-42.9	2.55	+0.19	5.42	22GY
A7	*qCaC.21NJ.A7*	82.61	Bn-A07-p20143131-Bn-A07-p19824864	81.9-84.7	3.73	-1.12	8.00	21NJ
A9	*qCaC.22NJ.A9*	30.31	Bn-A09-p30971818-Bn-A09-p32167322	28.5-34	3.13	-0.31	6.30	22NJ
C7	*qCaC.21NJ.C7-1*	16.91	Bn-scaff_16110_1-p3660807-Bn-scaff_16110_1-p3277472	16.6-18.9	2.64	-0.80	4.93	21NJ
C7	*qCaC.22GY.C7-2*	103.31	Bn-scaff_18202_1-p825947-Bn-scaff_21268_1-p102415	101.5-104	3.26	+0.19	6.96	22GY
C7	*qCaC.21GY.C7-3*	140.41	Bn-scaff_15627_1-p472369-Bn-scaff_16975_1-p113335	130.8-140.8	2.79	-0.33	8.27	21GY

Chr, chromosome; QTL, quantitative trait locus; Peak, physical locations of QTL peaks; Marker, Mark interval range; CL, physical locations on the chromosomes for the 95% confidence intervals of QTLs for CaC; LOD, logarithm of odds; ADD, additive effect, it is the accumulation of the genotype values of multiple micro-effect genes that affect quantitative traits, also known as the breeding value of a trait, and are the main component of the phenotypic value of a trait, the ‘+’ here represents from ‘APL01’, and the ‘-’ represents from ‘Holly’; R^2^: the phenotypic interpretation rate of the QTL.

### GWAS of the CF population

A GWAS of CF the population in six environments identified SNPs having -log10 (*P*) ≥ 5.16 as significant sites. A total of 282 significant SNPs were detected, and they were distributed on all of the chromosomes except A10 (**Supplementary Figure S1**). A total of 112 candidate association intervals were obtained by integrating these SNPs (**Supplementary Table S1**). Among them, 46 and 56 SNPs were distributed on A5 and C5 chromosomes, respectively, accounting for 16.3% and 19.9% of the total SNPs, respectively, but only two SNP sites were detected on both chromosomes A4 and A6. In addition, the number of SNPs significantly related to CaC in the six environments was quite different. There were only 13 SNPs detected in 22NJ, whereas 136 SNPs were identified in 21NJ.

Among the 112 candidate association intervals, 11 essential intervals were used for screening candidate genes (**Table 4**). In particular, the 43,898,035–44,350,778 (bp) interval on the A5 chromosome containing 38 SNPs and the 56,323,644–57,454,448 (bp) interval on C5 chromosome containing 44 SNPs, were used because it was speculated that they contained genes essential for the regulation of CaC. In addition, in the 23NJ and 21NJ environments, overlapping regions were found between 25,973,806 and 26,210,661 (bp) on chromosome A7.

**Table 4:** Screening candidate association intervals with a large number of SNPs through integration.

Chromosome	Physical position (bp)	Number of SNP	Experiments
A2	19087475-20887765	3	21NJ
A3	27295484-27703167	5	22GY
A5	45385651-45566391	5	21GY
A5	43898035-44350778	38	21NJ
A3	10453212-10727101	6	21NJ, 23NJ
A7	25973806-26210661	4	22NJ, 21GY
C3	5783333-5963981	5	21NJ
C3	14389343-14786128	7	21GY, 23GY
C4	66255834-66910039	13	21NJ
C4	67490825-67671425	2	22GY
C5	56323644-57454448	44	21NJ

### Co-localization combining the QTL and GWAS

The combined results of the QTL mapping and GWAS revealed that no duplicate sites were detected by the two methods. Chromosome A5 contained a major QTL, and the GWAS detected more SNP sites than on other chromosomes. Therefore, the A5 chromosome may be closely related to the CaC in *B. napus* shoots.

### Candidate gene prediction

**Figure 3:**
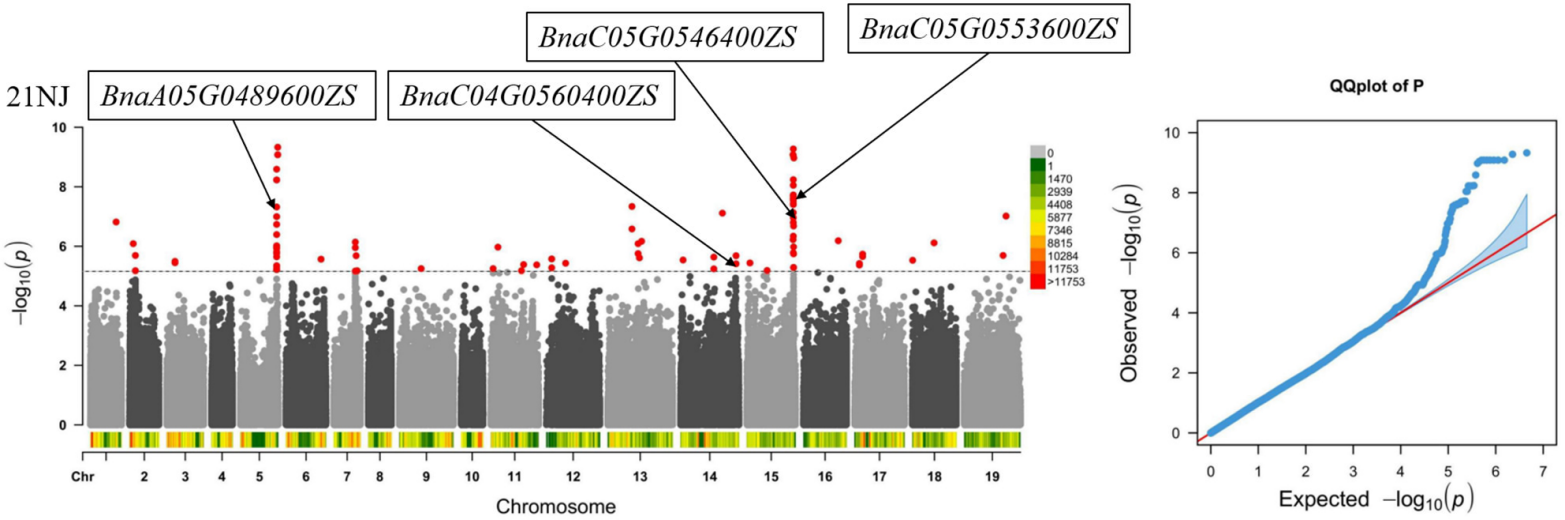
Genome-wide association study of the Ca concentration in the CF population. Identified candidate genes related to Ca concentration in the experimental environment 21NJ.

Based on the multiple sites involved in the absorption and transport of intracellular Ca detected by the QTL mapping and GWAS, combined with the genome model of *B. napus*, 10 major candidate genes were functionally annotated (**Table 5**). Based on the functional annotation of *Arabidopsis* homologs, there were four candidate genes related to CaC in the experimental environment 21NJ, which were mainly screened on chromosomes A5 and C5 (**Figure 3**).

**Table 5:** Candidate genes related to Ca concentration in *B. napus* shoots.

Gene name	Homologs	Chr	Physical position	Gene function
QTL				
*BnaC07g46260D*	MCU4	C7	43972670-43974228	mitochondrial calcium channel
*BnaC07g46310D*	MCU4	C7	43989764-43992718	mitochondrial calcium channel
*BnaC07g46660D*	ACA2	C7	44180545-44185434	calmodulin-regulated Ca^2+^-pump
*BnaA09g18100D*	AHA1	A9	11184831-11187154	reduced ability to close their stomata
GWAS				
*BnaA05G0489600ZS*	EXPA13	A5	44007868-44350778	plant-type cell wall loosening
*BnaC05G0546400ZS*	EXPA13	C5	56323644-56609571	plant-type cell wall loosening
*BnaC05G0553600ZS*	C2 calcium-lipid binding protein	C5	56757582-56982859	calcium and phospholipid-binding C2-domain
*BnaC04G0560400ZS*	CAX1	C4	67490825-67671425	calcium:cation antiporter activity
*BnaC03G0237200ZS*	CAM5	C3	14389343-14643998	calcium ion binding
*BnaA03G0493300ZS*	CBL3	A3	27295484-27476153	calcium ion binding

Among these 10 candidate genes, *BnaA05G0489600ZS* and *BnaC05G0546400ZS* homologous express the expansionA13 protein, which affects the formation of plant lateral roots (Wang et al., 2024). The candidate gene *BnaA09g18100D* uniformly expresses the H^+^-ATPase 1 protein (Yamauchi et al., 2016), which can regulate stomatal opening and affect plant transpiration. These proteins indirectly affect the absorption and transport of Ca by regulating other positions. The remaining five genes affected the redistribution of Ca^2+^ between cells by regulating the expression of vectors and channel proteins. *BnaC07g46260D* and *BnaC07g46310D* encode mitochondrial unidirectional cotransporter, a calcium ion transporter located on the mitochondrial membrane (Duan et al., 2024). *BnaC07g46660D* is homogeneously expressed with Ca ATPase 2, which is located in the organelle membrane system and is a transporter involved in Ca^2+^ regulation in plant cells (Rahmati Ishka et al., 2021). The homologous expression proteins of candidate gene *BnaC05G0553600ZS* belongs to the Ca lipid-binding family of proteins, which can bind Ca^2+^ and phospholipids involved in cell-membrane transport (Cui et al., 2023). *BnaC04G0560400ZS* encodes a homologous cationic 1 (CAX1) protein, which is highly correlated with Ca^2+^ ion accumulation. CAX1 shows a higher Ca^2+^ ion response in rapeseed (Navarro-León et al., 2019; Han et al., 2022). In addition, when the cell is under stress, CaC increases. *BnaC03G0237200ZS* and *BnaA03G0493300ZS* express the proteins of calmodulin 5 and calmodulin B-like 3, respectively, which bind to Ca^2+^ to signal and reduce ion concentrations in the cytoplasm (Ok et al., 2015).

## Discussion

The Ca, Se, and zinc contents in *B. napus* shoots are higher than in traditional vegetables (Fahey et al., 2001). Single pick-shoots have no significant effect on yield and can increase the grower’s economic benefits. As a green vegetable, the edible parts of *B. napus* are mainly the young stems and leaves at the stem elongation stage. The soluble solid contents and crude fiber levels of the AH and CF populations have been studied previously (Wu et al., 2021; Shi et al., 2023, Shi et al., 2024). Despite the limited understanding of the genetic mechanisms regulating CaC in *B. napus*, a combinatorial approach integrating QTL mapping and GWAS analysis has been employed to dissect the molecular basis of CaC at the genetic level.

### Phenotypic evaluation of group calcium content

Phenotypic data indicated that the average CaC of AH and CF population in the diverse environments were 7.41 and 6.17 mg/g, respectively, and ranged from 0.05 to 37.61 mg/g and 0.67 to 19.71mg/g, respectively (**Table 1**; **Table 2**). The mean values of CaC in different varieties of cabbage, cauliflower, and Chinese cabbage are 2.38, 1.89, and 3.30 mg/g, respectively, with ranges from 1.72 to 3.32 mg/g, 1.56 to 2.57 mg/g, and 2.83 to 3.89 mg/g, respectively (Yue et al., 2024). The dry matter minerals of beans, leafy vegetables, and grains have been determined, and leafy vegetables have the highest CaC. The average value for new cocoyam leaves is 6.5 mg/g, ranging from 6.05 to 6.82 mg/g, and the average value of Cassava leaves is 2.64 mg/g, ranging from 1.78 to 3.91mg/g (Castro-Alba et al., 2019). There lines in both the AH and CF populations that had much higher CaC than common vegetables. Therefore, there is an excellent potential to develop high-Ca OVDP varieties. In addition, there are high-quality germplasm resources in the two populations that can be used to cultivate new vegetables with higher CaC.

### Comprehensive analysis of group calcium content

At present, studies on Ca in vegetables mainly focus on comparing the CaC among different kinds of vegetables (Nerdy, 2018), the effect of Ca fertilizer on plant growth (Yuan et al., 2018), and Ca signaling between plant cells (Li et al., 2024). Few studies have been conducted to understand the influence of the CaC-related genes in crops by integrating QTL mapping and a GWAS, and only a few crops, such as wheat, pea, and tomato (Capel et al., 2017; Hussain et al., 2017; Ma et al., 2017), have been studied. In the present study, 12 QTLs were detected in the AH population, with PVs ranging from 1.67% to 10.10%, and one QTL located on chromosome A5 explained more than 10% of the total PV (**Table 3**). All of the 12 QTLs were environment-specific QTLs detected in a single environment. In addition, 282 significant SNPs were detected in the CF population, and 112 candidate association regions were obtained by integration (**Supplementary Table S1**). No duplicate sites were found in the two populations in different environments. The calcium content in B. *napus* is primarily derived from soil uptake, which involves the coordinated activity of multiple transmembrane transporters (e.g., CAX family). Given the substantial effects of environmental variables (e.g., soil pH, temperature, and nutrient availability) on both gene expression and ion bioavailability, the observed phenotypic variation in CaC across experimental environments reflects complex genotype-by-environment interactions (DalCorso et al., 2014; Wu et al., 2021). Here, utilizing a combined analysis to investigate the genetic mechanisms of CaC in *B. napus* shoots led to the identification of superior resources from genomics and also provided a new strategy for improving the quality of other vegetables.

### Functional prediction of candidate genes

Candidate genes for controlling mineral elements have been mined in other crops, but limited CaC-regulating genes have been identified in cruciferous vegetables. Based on the Ca transport process Ca in plant cells and the functional annotations of candidate genes, this research explored the candidate genes that control CaC. Finally, 10 CaC-related candidate genes were screened, and the homologous proteins encoded by these genes regulated CaC by affecting the Ca uptake rate, upward transport process, and inter-tissue Ca^2+^ allocation in rapeseed. The candidate genes *BnaA05G0489600ZS* and *BnaC05G0546400ZS* were homologous to *expansionA13*, which encodes a protein that loosens cell walls. Overexpression of this protein in *Arabidopsis* increases the number of lateral roots formed (Lee and Kim, 2013), a well-developed root structure can improve the Ca absorption rate of rapeseed. According to the functional annotation, *BnaA09g18100D* encodes the H^+^-ATPase 1 protein that regulates stomatal opening, which controls the bottom-up transport of Ca in rapeseed by influencing plant transpiration (Yamauchi et al., 2016). In addition, Ca mainly exists in the form of ions and enters plant cells through carrier and channel proteins. Among the 10 candidate genes, 5 genes were annotated as carrier proteins, which were related to the CAX1, Ca lipid-binding, mitochondrial unidirectional cotransporter, and Ca ATPase 2 proteins. Homologous proteins of two candidate genes are involved in the regulation of Ca^2+^ concentration in the cytoplasm. In other crops, such as *Arabidopsis*, cotton, and tomato, these proteins transport Ca^2+^ into cellular tissues under response conditions, binding with proteins, pectin, phosphate, and other components. *BnaC04G0560400ZS* is a vital candidate gene, and its encoded protein, CAX1, can transport Ca^2+^ ions to vacuoles for storage and accumulation (Han et al., 2022). In carrots, this protein improves Ca bioavailability and promotes growth (Morris et al., 2006), but there are limited studies in vegetables. Therefore, this protein will provide a basis for research on breeding high-Ca *B. napus* varieties. The functions of these candidate genes need to be further verified. Still, this research helped increase the understanding of the genetic mechanisms behind the CaC in *B. napus* and provides a reference for breeding high-quality vegetable rapeseed varieties.

## Data Availability

The original contributions presented in the study are included in the article/**Supplementary Material**. Further inquiries can be directed to the corresponding author/s.

## Supplementary material

Supplementary Table 1 Integration of all the relevant SNP’s Ca concentrations from *B. napus* shoots in six distinct environments.

Supplementary Figure 1 Genome-wide association study of the Ca concentration of the CF population. **(A)** Manhattan map (left) and QQ map (right) of the CF population planted in the Guiyang area in 2021; **(B)** Manhattan map (left) and QQ map (right) of the CF population planted in the Nanjing area in 2021; **(C)** anhattan map (left) and QQ map (right) of the CF population planted in the Guiyang area in 2022; **(D)** Manhattan map (left) and QQ map (right) of the CF population planted in the Nanjing area in 2022; **(E)** Manhattan map (left) and QQ map (right) of the CF population planted in the Guiyang area in 2023; **(F)** Manhattan map (left) and QQ map (right) of the CF population planted in the Nanjing area in 2023.
