# Supplementary material for: Integrating QTL mapping and GWAS to decipher the genetic mechanisms behind the calcium contents of Brassica napus shoots
Source: Front Plant Sci. 2025 Apr 10;16:1565329. doi: 10.3389/fpls.2025.1565329 (PMC12018428; doi:10.3389/fpls.2025.1565329)
Supplement: Supplementary Table 1 — Integration of all the relevant SNP’s Ca concentrations from B. napus shoots in six distinct environments. [file Table1.docx]

**TABLE S1:** **Integration of all the relevant SNP’s Ca concentrations from *B. napus* shoots in six distinct environments.**

| **Chromosome** | **Physical position** (bp) | **Number of SNP** | **Experiments** |
| --- | --- | --- | --- |
| scaffoldA01 | 7993253-8173853 | 1 | 23GY |
| scaffoldA01 | 11459336-12061646 | 2 | 21GY, 22GY |
| scaffoldA01 | 23186238-23366838 | 1 | 23NJ |
| scaffoldA01 | 31037481-31218081 | 1 | 21NJ |
| scaffoldA02 | 4377095-4557695 | 1 | 21NJ |
| scaffoldA02 | 6980934-7293854 | 3 | 21NJ |
| scaffoldA02 | 17893020-18073620 | 1 | 22GY |
| scaffoldA02 | 19087475-20887765 | 3 | 22NJ, 23NJ, 21GY |
| scaffoldA03 | 7668069-7848669 | 1 | 23GY |
| scaffoldA03 | 9711023-9891627 | 3 | 21NJ |
| scaffoldA03 | 10453212-10727101 | 6 | 23NJ |
| scaffoldA03 | 16333709-16514309 | 1 | 21GY |
| scaffoldA03 | 22134506-22315106 | 1 | 22NJ |
| scaffoldA03 | 25684439-25865056 | 3 | 23NJ |
| scaffoldA03 | 27295484-27703167 | 5 | 22GY |
| scaffoldA03 | 28981004-29161604 | 1 | 21GY |
| scaffoldA03 | 33307155-33487755 | 1 | 23NJ |
| scaffoldA03 | 37841449-38022049 | 1 | 21GY |
| scaffoldA03 | 42759626-42940226 | 1 | 22NJ |
| scaffoldA04 | 13665335-13845935 | 1 | 23NJ |
| scaffoldA04 | 25191228-25371828 | 1 | 23NJ |
| scaffoldA05 | 2374072-2670061 | 2 | 22GY |
| scaffoldA05 | 42622769-42803369 | 1 | 22NJ |
| scaffoldA05 | 43898035-44350778 | 38 | 21NJ |
| scaffoldA05 | 45385651-45566391 | 5 | 21NJ |
| scaffoldA06 | 42466356-42646968 | 2 | 21NJ |
| scaffoldA07 | 1323047-1503669 | 3 | 21GY |
| scaffoldA07 | 8696906-8877506 | 1 | 22GY |
| scaffoldA07 | 11126321-11306921 | 1 | 21GY |
| scaffoldA07 | 25973806-26210661 | 4 | 21NJ, 23NJ |
| scaffoldA07 | 26937848-27118448 | 1 | 21NJ |
| scaffoldA07 | 28335541-28525768 | 2 | 21NJ, 22GY |
| scaffoldA08 | 7740356-7920956 | 1 | 23NJ |
| scaffoldA08 | 13927562-14108162 | 1 | 22GY |
| scaffoldA08 | 23068963-23249568 | 2 | 23GY |
| scaffoldA08 | 25189710-25898803 | 3 | 22NJ, 21GY |
| scaffoldA09 | 9138626-9319226 | 1 | 23GY |
| scaffoldA09 | 21788506-21969106 | 1 | 22GY |
| scaffoldA09 | 26365937-26546537 | 1 | 21NJ |
| scaffoldA09 | 63135364-63490465 | 2 | 22NJ, 21GY |
| scaffoldC01 | 1174627-1449470 | 2 | 22NJ |
| scaffoldC01 | 2483042-2663642 | 1 | 21NJ |
| scaffoldC01 | 8379645-8560245 | 1 | 21NJ |
| scaffoldC01 | 10160844-10341444 | 1 | 21GY |
| scaffoldC01 | 13676291-14148965 | 2 | 21GY, 23GY |
| scaffoldC01 | 20176702-20357302 | 1 | 21GY |
| scaffoldC01 | 31506654-31687495 | 4 | 21GY |
| scaffoldC01 | 37169373-37349973 | 1 | 21NJ |
| scaffoldC01 | 39579863-39760463 | 1 | 21NJ |
| scaffoldC01 | 53412038-53592638 | 1 | 23GY |
| scaffoldC01 | 55585964-55766564 | 1 | 21NJ |
| scaffoldC02 | 6528063-6708990 | 3 | 21NJ |
| scaffoldC02 | 16891146-17071746 | 1 | 23NJ |
| scaffoldC02 | 23477808-23658408 | 1 | 21NJ |
| scaffoldC02 | 33541459-33722059 | 1 | 21GY |
| scaffoldC02 | 50838199-51018807 | 2 | 23NJ |
| scaffoldC03 | 5783333-5963981 | 5 | 21GY |
| scaffoldC03 | 14389343-14786128 | 7 | 23NJ, 22GY, 23NJ |
| scaffoldC03 | 24197472-24378072 | 1 | 23GY |
| scaffoldC03 | 29505684-29686289 | 2 | 21NJ |
| scaffoldC03 | 36876940-37057554 | 2 | 21NJ |
| scaffoldC03 | 38507617-38688217 | 1 | 21NJ |
| scaffoldC03 | 41031157-41211757 | 1 | 21NJ |
| scaffoldC03 | 44685878-44866478 | 1 | 21GY |
| scaffoldC03 | 58603085-58783685 | 1 | 23NJ |
| scaffoldC03 | 63887107-64067707 | 1 | 23GY |
| scaffoldC04 | 2833902-3014502 | 1 | 21NJ |
| scaffoldC04 | 12184676-12365276 | 1 | 22GY |
| scaffoldC04 | 21125179-21769867 | 3 | 22GY, 22NJ |
| scaffoldC04 | 22714173-22894773 | 1 | 23GY |
| scaffoldC04 | 38866962-39047567 | 3 | 21GY |
| scaffoldC04 | 40552591-40733194 | 2 | 21NJ |
| scaffoldC04 | 50826282-51006882 | 1 | 21NJ |
| scaffoldC04 | 65008908-65189509 | 2 | 23GY |
| scaffoldC04 | 66255834-66910039 | 13 | 21GY, 23GY |
| scaffoldC04 | 67490825-67671425 | 2 | 21NJ |
| scaffoldC05 | 3573842-3754442 | 1 | 21NJ |
| scaffoldC05 | 12473940-12654540 | 1 | 23GY |
| scaffoldC05 | 16935169-17115769 | 1 | 22NJ |
| scaffoldC05 | 24402087-24605331 | 2 | 23NJ, 21NJ |
| scaffoldC05 | 34623468-35250625 | 2 | 22GY |
| scaffoldC05 | 36359593-37432717 | 3 | 21GY |
| scaffoldC05 | 45148908-45329508 | 1 | 21GY |
| scaffoldC05 | 48156529-48337129 | 1 | 21GY |
| scaffoldC05 | 56323644-57454448 | 44 | 21NJ |
| scaffoldC06 | 28158921-28339521 | 1 | 21GY |
| scaffoldC06 | 42271232-42451832 | 1 | 21NJ |
| scaffoldC06 | 45243940-45424545 | 2 | 22GY |
| scaffoldC06 | 47641695-47822304 | 3 | 22GY |
| scaffoldC07 | 1776896-1957496 | 1 | 22NJ |
| scaffoldC07 | 5804438-5985040 | 2 | 21NJ |
| scaffoldC07 | 9271444-10008013 | 3 | 22GY, 21NJ |
| scaffoldC07 | 19353163-20386333 | 3 | 22GY, 23GY |
| scaffoldC07 | 30667695-30848295 | 1 | 23GY |
| scaffoldC07 | 41249678-41430278 | 1 | 21GY |
| scaffoldC07 | 54499008-54679608 | 1 | 21GY |
| scaffoldC08 | 250338-435416 | 2 | 21NJ, 23GY |
| scaffoldC08 | 17664462-17845062 | 1 | 23GY |
| scaffoldC08 | 23470419-23651019 | 1 | 22GY |
| scaffoldC08 | 26292334-26858587 | 3 | 21NJ, 21GY |
| scaffoldC08 | 33236131-34425978 | 2 | 21GY, 22NJ |
| scaffoldC08 | 35361099-35541699 | 1 | 21GY |
| scaffoldC08 | 40095103-40275703 | 1 | 23NJ |
| scaffoldC09 | 12164099-12344699 | 1 | 21GY |
| scaffoldC09 | 20956899-21137499 | 1 | 21GY |
| scaffoldC09 | 24597107-24777707 | 1 | 23NJ |
| scaffoldC09 | 47087404-47268004 | 1 | 21NJ |
| scaffoldC09 | 48573481-48754081 | 1 | 21GY |
| scaffoldC09 | 50775866-50956473 | 2 | 21NJ |
| scaffoldC09 | 53814164-53994764 | 1 | 23GY |
| scaffoldC09 | 57478343-57658943 | 1 | 22GY |
| scaffoldC09 | 65429756-65610356 | 1 | 23GY |
